# What is new in the liver sinusoids? meeting report, 16th International Symposium on Cells of the Hepatic Sinusoid (ISCHS)

**DOI:** 10.1186/1755-1536-4-27

**Published:** 2011-12-13

**Authors:** Krista Rombouts, Massimo Pinzani

**Affiliations:** 1 Department of Internal Medicine, DENOThe, University of Florence, Florence, Italy

## Abstract

The 16th International Symposium on Cells of the Hepatic Sinusoid (ISCHS) took place in Florence, Italy on 22-24 September 2011. This symposium is a multidisciplinary meeting where new and important findings on the biology of liver cells are presented and discussed.

## Introduction

The 16th International Symposium on Cells of the Hepatic Sinusoid (ISCHS) took place in Florence, Italy on 22-24 September 2011 and was attended by 150 scientists from 16 Nations. This was the second meeting of the newly formed International Society for Hepatic Sinusoidal Research (ISHSR). The first meeting was held last year in Pasadena, California (USA) and originates from the historical series of ISCHS, founded in 1977.

This year, the meeting showcased two state-of-the-art lectures: *Cholangiocytes and angiogenesis *by Mario Strazzabosco (University of Milan-Bicocca, Milan, Italy and Yale University Liver Center, New Haven, CT, USA), and *The functional role of chemokines in different chronic liver diseases (CLD): focus on sinusoidal cells *by Herman Wasmuth (University Hospital Aachen, Aachen, Germany). Two tutorial lectures were given concerning 'methods in hepatology' that covered new findings in the field of microRNAs and hepatic stellate cells (HSC) presented by Norifumi Kawada (Osaka City University, Osaka, Japan), and methods for the study of neoangiogenesis in liver diseases presented by David Semela (University Hospital Basel, Basel, Switzerland).

The meeting was divided into seven thematic sessions: (1) sinusoidal cells and the regulation of hepatic inflammation, (2) nuclear receptors and liver cells, (3) sinusoidal cells in alcoholic and non-alcoholic steatohepatitis (ASH and NASH), (4) sinusoidal cells in metabolism, portal hypertension, ischemia-reperfusion injury and graft preservation, (5) sinusoidal cells, inflammation and the microbiota, (6) nanotechnologies and advanced therapeutics, and (7) role of sinusoidal cells (liver stroma) in primary and metastatic liver cancer. All eight sessions were covered by 18 lectures given by experts in a specific topic followed by a total of 23 oral presentations presented by young investigators. Two selected poster overview sessions were dedicated to posters presented at the meeting that represented excellent, well designed and innovative topics related to all sessions covered during this meeting. The symposium also honored one of our colleagues who recently died. Massimo Pinzani and Gianluca Svegliati-Baroni gave a short commemoration of Professor Marcos Rojkind (1935-2011), Professor of Biochemistry, Molecular Biology and Pathology at George Washington University Medical Center, Washington DC, USA. This year, the work of Le Thi Thanh Thuy (Osaka City University, Osaka, Japan) was awarded with the Marco Foschi Prize for Research in Gastrointestinal Oncology. This prize was assigned to a young researcher for the best ISHSR 2011 oral presentation in the area of oncology.

This meeting was sponsored by ISHSR and cosponsored by the European Association for the Study of the Liver (EASL), American Association for the Study of Liver Diseases (AASLD), the National Institutes of Health, USA (NIAAA), and industrial support was given by Pfizer, Merck & Co. and Intercept Pharmaceuticals.

### Session 1: sinusoidal cells and the regulation of hepatic inflammation

The first invited lecture in this session was given by Percy Knolle (Institute of Molecular Medicine and Immunology, University of Bonn, Bonn, Germany), introducing the important link between the innate immune system and the adaptive immunity in the liver. He introduced the unique immune functions to regulate local and systemic immunity, with the engagement of HSC and liver sinusoidal endothelial cells (LSEC) in the regulation of the local immunity system. Percy demonstrated that LSEC have immune cell characteristics because they crossprime naïve CD8 T cells for tolerance or immunity.

Nicholas Shackel (AW Morrow Gastroenterology and Liver Centre, Camperdown, Australia), presented a novel role of CD147, an abundant membrane receptor glycoprotein ubiquitously expressed on hepatocytes and liver leukocytes, in the context of intrahepatic inflammation. Liver injury was induced in murine models, that is C57BL/6, BALB/c and CD147^-/- ^mice, with either thioacetamide (TAA) or CCl_4_. The authors of this work demonstrated that liver leukocytes undergo CD45+ immune cell aggregates in progressive liver injury irrespective of cause of injury. This contributes directly to the magnitude of injury with progressive inflammation-associated liver injury.

Tumor necrosis factor α (TNFα)-converting enzyme (TACE) is key to produce active TNFα, and is negatively regulated by tissue inhibitor of metalloproteinases 3 (TIMP3), which is under the control of the silent information regulator 1 (Sirt1). Nathalie Török (Division of Gastroenterology and Hepatology, UC Davis, Sacramento, CA, USA) demonstrated that advanced glycation end products modulate proinflammatory activity by inducing TACE activity *in vitro *and *in vivo *in a NASH model. To assess the Sirt1/TIMP3/TACE axis *in vivo*, mice were fed by normal chow, choline-supplemented, L-amino acid (CSAA) or choline-deficient, L-amino acid (CDAA) diets. The effect of TIMP3 on TACE activity was quantified by injecting a TIMP3-expressing adenoviral vector. This resulted in a decrease in TACE and profibrogenic activity.

The second invited lecture in this session, presented by Bin Gao, (NIAAA, NIH, Bethesda, MD, USA) addressed the link between the innate immune system and the liver tissue repair process. The liver relies on its strong innate immune system to effectively and quickly defend against potentially toxic agents, originating from the blood, without launching harmful immune responses. This comprises enrichment of innate immune cells, removal of waste molecules and immunologic elimination of microorganisms by liver endothelial cells and Kupffer cells. Recent advances on the diverse roles of natural killer and natural killer T cells in hepatocellular damage, liver fibrogenesis, and liver regeneration were further discussed.

Additional and very relevant evidence that hepatitis C virus (HCV) proteins can directly affect liver inflammation and fibrosis was presented by Soichi Kojima (RIKEN ASI, Saitama, Japan). The authors of this work demonstrated that HCV NS3 protease mimics transforming growth factor (TGF)β2 and activates TGFβ signals via type I receptor. The results indicated that NS3 protease possesses the immunogenic and biological activities of TGFβ2. Antibodies that recognized the three binding sites necessary for the interaction between NS3 and TGFβ receptor I blocked the TGFβ activity mimicked by NS3.

### Session 2: nuclear receptors and liver cells

The first invited lecture in this session was given by Michael Trauner (Medical University of Vienna, Vienna, Austria), who addressed the functional role of the large family of nuclear receptors (NRs) in liver and liver diseases. As metabolic sensors, NRs are important in processes that involve endobiotics and xenobiotics, regulating the homeostasis of metabolism. Changes at the transcriptional level lead to NR-deficient function and can result in liver diseases acting on multiple cellular levels affecting transport, metabolism, inflammation and fibrosis. Therefore, the search to target this NR led to several molecular compounds to treat cholestasis and non-alcoholic fatty liver disease (NAFLD).

In the second invited lecture Luciano Adorini (Intercept Pharmaceuticals, New York, NY, USA) gave an overview of the working mechanism of bile acids that activate two NRs: farnesoid × receptor (FXR) and the G-protein-coupled receptor TGR5. These receptors are interesting targets for a variety of diseases affecting liver, kidney and intestine, in addition to metabolic diseases. Moreover, new clinical data were shown on the use of the FXR agonist INT-747, which has been successfully tested in clinical studies, showing efficacy in patients with primary biliary cirrhosis, type 2 diabetes and NAFLD.

The application of targeting a NR with specific ligands was further demonstrated by V Balasubramaniyan (Institute of Hepatology, University College London, London, UK). The activation of FXR by its agonist INT-747 restores hepatic endothelial nitric oxide synthase (NOS) activity and lowers portal pressure in bile duct ligation (BDL)-induced cirrhotic rats by modulating the dimethylarginine dimethylaminohydrolase 1 (DDAH)-asymmetric dimethyarginine (ADMA) pathway. DDAH-2 expression was reduced and this coincided with a significant lowering of portal pressure. These data provide further evidence to support the use of specific agonists such as INT-747 to restore FXR hepatoprotective function in liver diseases that are characterized by portal hypertension.

That further research is needed to examine the ligand specificity of the agonist INT-747 was shown by Matthew Wright (Newcastle University, Newcastle, UK), who demonstrated that INT-747 is also an activator of the pregnane × receptor (PXR), another rather promiscuous nuclear receptor that binds several bile acids. Administration of INT-747 to pxr^+/+ ^mice induced hepatic expression of PXR-inducible cyp3a11 and cyp3a25 mRNAs. Hepatic induction of cyp3a11 and cyp3a25 was abolished in mice with a disrupted pxr gene. In this work, the authors showed that the FXR agonist INT-747 also activates PXR in both human and mouse.

### Session 3: sinusoidal cells in alcoholic and non-alcoholic steatohepatitis

As first invited lecturer, Isabelle Leclercq (Université Catholique de Louvain, Brussels, Belgium) gave an excellent overview of the very complex process of insulin resistance (IR) and its impact on the liver and peripheral tissues. Isabelle explained that IR is defined by the impaired responsiveness of target organs to circulating insulin translated as impaired inhibition of hepatic glucose production, decreased peripheral glucose uptake, leading to hyperglycemia, hyperinsulinemia and evolution towards type 2 diabetes, and increased free fatty acids released by adipose tissue.

Ariel Feldstein (University of California, San Diego, CA, USA) addressed lipotoxicity and related effects on sinusoidal cells in the second invited lecture of this session. NAFLD, the hepatic component of the metabolic syndrome, is a highly prevalent condition and associated with increased morbidity and mortality. Studies have uncovered a novel pathogenic role for adipose tissue macrophages and Kupffer cells in the pathogenesis of hepatic steatosis. This lecture showed that lipid partitioning and lipotoxicity have emerged as key pathogenic mechanisms in liver damage and NASH development.

That HSC play a key role in alcoholic hepatitis (AH) was proven by Silvia Affò (Liver Unit, IDIBAPS, Barcelona, Spain). The investigator demonstrated that chemokine (C-C motif) ligand 20 (CCL20) is a novel mediator of inflammation and fibrosis in AH due to CCL20 expression in HSC. The upregulation of hepatic CCL20 gene expression and serum levels was specific for AH patients. By performing *in vitro *experiments in HSC, the authors demonstrated that lipopolysaccharide (LPS), TNFα and interleukin 1β (IL-1β) induced CCL20 gene expression and CCL20-induced fibrogenic markers, wound healing and migration.

Wheatly Antony (University of Otago, Dunedin, New Zealand) provided new insights into concanavalin A (ConA)-induced hepatitis and the relative roles of gut microflora, intestinal permeability, and Toll-like receptor 4 (TLR4). ConA administration was tested in TLR4^-/-^, C3H/HeN (LPS-sensitive) and C3H/HeJ (LPS-insensitive) mice, and in C57/BL6 mice pretreated with the anti-TLR4 monoclonal antibody (mAb), MTS510. Overall, the results of this study indicate that ConA-induced hepatitis is marked by increased intestinal permeability and requires the presence of a functional TLR4 and of Gram-negative bacteria or their products (LPS) in the gut. ConA administration increased the percentage of TLR4-positive CD3^+^-T cells and Kupffer cells.

Information on the role of liver dendritic cells (DC) in the pathogenesis of ethanol-induced steatosis in an acute alcoholic liver injury (AALI) model in mice was provided by Costica Aloman (Mount Sinai School of Medicine, New York, USA). DC development is regulated especially by Fms-like-3 tyrosine kinase ligand (Flt3L) and its receptor Flt3. The authors induced AALI in Flt3L-treated mice, in conventional dendritic cell (cDC)-depleted CD11c-DTR mice that express the diphtheria toxin receptor and in Flt3 knockout (Flt3-KO) mice. The overall outcome of this study indicates a key role for cDC-controlled development of steatosis during AALI.

Kenichi Ikejima (Juntendo University School of Medicine, Tokyo, Japan) demonstrated that mice depleted of natural killer T (NKT) cells are more susceptible to dietary-induced steatohepatitis. CD1d-KO animals, which lack mature NKT cells, and wild-type (WT) mice were fed a high-fat diet (HFD). Mice depleted of NKT cells appear to be more susceptible to HFD-induced steatohepatitis, indicating that hepatic NKT cells play a protective role against development of dietary steatohepatitis. This involves a downregulation of NKT cell-derived T helper 2 (Th2) cytokines, a decreased synthesis of apolipoprotein B/very low density lipoprotein (VLDL) and β-oxidation. Therefore, NKT cells modulate hepatic lipid metabolism, thus controlling the susceptibility to NASH.

### Session 4: sinusoidal cells in metabolism, portal hypertension, ischemia-reperfusion injury and graft preservation

The first invited lecture in this session was given by Bard Smedsrod (University of Tromsø, Tromsø, Norway) describing the sinusoidal endothelial cells and metabolic control. Bard demonstrated that LSEC play a central metabolic role by taking up and catabolizing a large number of molecules. The cells are geared to efficient receptor-mediated endocytosis and contain high lysosomal enzyme activity, giving the cells a very high capacity to catabolize endocytosed material. LSEC operate via a largely anaerobic metabolism because studies have demonstrated that the cells release large amounts of lactate and acetate, do not utilize glucose, carry few mitochondria, and are located in a low oxygen environment.

As a result of the scarce information so far available on the physiological function of human LSEC (hLSEC), Cristina Øie (University of Tromsø, Tromsø, Norway) set out to isolate and culture human LSEC. The author studied the expression of endocytic receptors and other phenotypic markers, and their ability to perform active endocytosis via these receptors. hLSEC maintain phenotypic uniqueness, an appropriate profile for scavenger receptors and endocytic function in culture, which was preserved at early cell passages but reduced with later passages, and this was accompanied by loss of specific phenotypic LSEC markers.

Giusi Marrone (Hospital Clínic-IDIBAPS and CIBEREHD, Barcelona, Spain) investigated the role of the transcription factor Kruppel-like factor 2 (KLF2), which mediates hepatic endothelial protection conferred by statins. In this study the effect of statins on KLF2 functions within the liver was investigated. Hepatic endothelial cells were treated with different statins, in the presence of the inhibitor mevalonate, under static or controlled shear stress conditions. Statin treatment induced systemic or liver endothelial KLF2 upregulation, which was inhibited by mevalonate, and this was confirmed by *in vivo *experiments. These data reinforce the therapeutic potential of these drugs for liver diseases.

The second invited lecturer, Maurizio Parola (Experimental Medicine and Oncology, University of Torino, Turin, Italy) addressed hypoxia-dependent and independent angiogenesis. Maurizio explained that hepatic angiogenesis is involved in the progression of experimental and clinical CLD and is believed to favor fibrogenesis since experimental antiangiogenic strategies can effectively block or attenuate fibrosis. Although hypoxia and hypoxia-related mechanisms are key in mediating hepatic angiogenesis in CLD, hypoxia-independent angiogenesis also plays a significant role with a major involvement of redox reactions and reactive oxygen species. Hepatic myofibroblasts have emerged as a putative critical crossroads between hypoxia-dependent and independent angiogenesis because of their dual role as profibrogenic and proangiogenic cells.

Jonel Trebicka (University of Bonn, Bonn, Germany) reported that statins induce senescence in activated HSC and interfere with angiogenesis in liver cirrhosis and portal hypertension. In this study cirrhosis was induced by BDL and CCl_4 _intoxication and portal hypertension was induced by partial portal vein ligation. Results show that statin therapy is beneficial in liver cirrhosis because it decreases portal pressure, induces senescence in activated HSC, decreases collateral formation, and activates hedgehog signaling in vascular smooth muscle cells (VSMC). The author notes that statins might aggravate collateral formation and even increases portal pressure in non-cirrhotic portal hypertension.

Chiara Busletta (University of Torino, Turin, Italy) investigated the effect of hypoxia and oncostatin M (OSM) on human mesenchymal stem cells (hMSC) and found that both conditions exert motogenic stimulus on profibrogenic cells through a biphasic mechanism. This mechanism is characterized by a first phase switched on by rapid generation of reactive oxygen species (ROS), whereas late migration is sustained by hypoxia-inducible factor 1 (HIF-1)-dependent expression and release of vascular epithelial growth factor (VEGF). OSM operates through mechanisms homologous to those elicited by hypoxia. Hypoxia-dependent and OSM-dependent migration were abolished by rotenone or apocynin, indicating mitochondria or nicotinamide adenine dinucleotide phosphate (NADPH) as a source of ROS, respectively.

### Session 5: sinusoidal cells, inflammation and the microbiota

The invited lecture of this session was given by Gianluca Svegliati-Baroni (Università Politecnica delle Marche, Ancona, Italy), addressing the microbiota in steatohepatitis. Gianluca demonstrated the direct correlation between gut microbiota and obesity as obese individuals have a different microbiota composition in comparison to lean individuals. Furthermore, data showed that small intestinal bacterial overgrowth and consequent bacterial translocation are strictly related to hepatic fibrogenesis and its degeneration. Toll-like receptors and bacterial endotoxemia are involved in the process of liver fibrogenesis.

Laura Dixon (Case Western Reserve, Cleveland, OH, USA) reported that inflammasome-mediated caspase 1 activation in macrophages contributes to inflammation and fibrosis in diet-induced steatohepatitis. The role and cell specificity of caspase 1 activation in NASH was investigated. Caspase 1-KO mice were placed on a methionine and choline deficient (MCD) diet. Caspase 1-associated ASC ('apoptosis-associated speck-like protein containing a C-terminal caspase recruitment domain') was increased in MCD-fed mice indicating the hepatic inflammasome activation. The author showed that caspase 1 expression, F4/80 and Sirius red staining were significantly reduced in clodronate-treated MCD-fed mice, and this confirmed the coinvolvement of macrophages/Kupffer cells. These data demonstrate that caspase 1 activation plays a role in MCD-induced NASH.

Patricia Lalor (University of Birmingham, Birmingham, UK) reported that vascular adhesion protein 1 (VAP-1) modulates glucose and lipid uptake in NAFLD. The author showed an elevated level of VAP-1 in diseased liver. VAP-1 substrates and inhibitors were used to perform standard *ex vivo *radiolabelled glucose and fatty acid uptake assays, and showed that the VAP-1 semicarbazide-sensitive amine oxidase activity (SSAO) results in enhanced hepatic lipid and glucose uptake and changes in transporter expression. The author demonstrated that in human NAFLD global alterations occur in cellular expression of glucose and lipid transporter proteins.

### Session 6: sinusoidal cells in ASH and NASH

In this session the focus was on the effect of ethanol. The first invited lecture was given by Sam Zakhari (NIAAA, Bethesda, MD, USA), which introduced alcohol metabolism in the hepatic sinusoid and its role in liver disease. Ethanol metabolism increases ROS that leads to HSC activation, and superoxide anion release in sinusoids activates Kupffer cells. Alcohol-induced inflammation results in leukocyte trafficking through the sinusoids and the rate of leukocyte recruitment and the nature of recruited cells determines the severity of liver damage. Alcohol produces pre-sinusoid venoconstriction, probably due to prostaglandins produced from arachidonic acid metabolism by cytochrome P450 2E1.

The second invited lecturer of this session, Gyongyi Szabo (University of Massachusetts Medical School, Waltham, MA, USA) discussed the effect of ethanol and signaling modulation in liver non-parenchymal cells and further emphasized on the role of TLR4-dependent signaling pathways that mediate alcohol-induced liver steatosis and inflammation. Kupffer cell sensitization is mediated via alcohol-induced upregulation of microRNA-155 that increases the message stability of TNFα. There is activation of the inflammasome complex in liver mononuclear cells in both ALD and NAFLD. These results suggest that activation of innate immune signaling extends to both TLR-induced and inflammasome-mediated activation of the inflammatory cascade in ALD.

Hidekazu Tsukamoto (University of Southern California School of Medicine, Los Angeles, CA, USA) discussed second and multiple-hit models of ALD. Gene and environmental interactions underlie chronic diseases including ALD. The involvement of interactions among lifestyle factors i.e., viral hepatitis and alcohol, obesity and alcohol, or triple combination, is becoming clinically apparent. They induce synergistic effects on the genesis of fatty liver, steatohepatitis, and liver cancer. Accordingly, animal models that reflect these synergistic pathologic outcomes are urgently needed for investigation of molecular mechanisms. Hide gave several examples of animal models where molecular mechanisms had identified potential new therapeutic targets for synergistic alcohol-associated liver diseases.

Fatima Teixeira-Clerc (INSERM, Paris, France) provided information on the mechanism by which the cannabinoid CB2 receptor exerts anti-inflammatory effects in Kupffer cells during ALD. These data demonstrate that the CB2 receptor displays beneficial effects on alcohol-induced inflammation by inducing heme oxygenase 1 (HO-1) in Kupffer cells, thereby limiting nuclear factor κB (NFκB) activation and the proinflammatory M1 response. Alcohol-fed mice were treated with the CB2 agonist JWH-133 and showed increased expression of HO-1 protein in Kupffer cells. HO-1 inhibition also prevented the inhibitory effect of JWH-133 on LPS-induced IL-6 and IL-1β expressions. These new data further support the CB2 receptor as a new anti-inflammatory target in the management of ALD.

Michele Pritchard (Cleveland Clinic, OH, USA) identified the protective effects of adenosine signaling in ethanol-induced liver injury, which may be a useful anti-inflammatory pathway to target and to attenuate the pathophysiological effects of ethanol in the liver. Here, the authors showed that adenosine, acting via the adenosine 2a receptor (A2AR), suppresses inflammatory cytokine expression by Kupffer cells and that the anti-inflammatory function of HO-1/globular adiponectin (gAcrp) also involves the adenosine-mediated signaling pathway. *In vivo *data showed that mice fed a Lieber-deCarli ethanol diet treated with cobalt protoporphyrin (CoPP) or an A2AR agonist increased both hepatic HO-1 and A2AR mRNA expression in mice.

### Session 7: nanotechnologies and advanced therapeutics

The first lecture was given by Mark Kester (Penn State Hershey Cancer Institute, Hershey, PA, USA), which introduced the clinical potential of sphingolipid-based nanotherapeutics. Several examples were given concerning the use of nanoconstructs to specifically target those genes in a cell in a specific way in diseases. Stable, non-toxic nanoscale formulations of ceramide have been engineered, including nanoliposomes, nanocolloids and nanodendrimers. Mark demonstrated that these 'nanosolutions' reduce *in vivo *tumor burden in models of hepatocellular carcinoma (HCC), and breast/pancreatic/melanoma cancer as well as leukemias. Nanotechnology has the potential to 'deliver' the promise of ceramide-based pharmaceutics.

Klaas Poelstra (University of Groningen, Groningen, The Netherlands) introduced a system of how to target drug delivery, and focused on the HSC as key player during liver fibrosis. Cell-specific drug delivery to HSC improves their therapeutic efficacy, which is particularly important for antifibrotic agents. Furthermore, drug targeting can also be applied to examine the relative contribution of a particular pathway, or cytokine, in disease progression. Klaas showed new data of how interferon γ (IFNγ) and IL-10 can be delivered to platelet-derived growth factor β (PDGFβ) receptors, which are highly expressed by HSC. This leads to a significant improvement of their safety profile and efficacy and opens up the possibility of a clinical application.

Maria Lauda Tomasi (USC Research Center for Liver Diseases, Los Angeles, CA, USA) reported that S-adenosylmethionine (SAMe) regulates ubiquitin-conjugating enzyme 9 (Ubc9) expression and sumoylation, with implications in hepatocarcinogenesis and treatment. Methionine adenosyltransferase 1a-KO mice (Mat1a-KO) are characterized by hepatic SAMe deficiency and intragastric ethanol-fed mice are marked by an increased susceptibility to steatosis and oxidative liver injury, spontaneous development of steatohepatitis and HCC. Ubc9 expression and sumoylation are increased when SAMe levels fall and they contribute to many of the abnormalities seen in both Mat1a-KO and alcohol-fed mice. SAMe and its metabolite methylthioadenosine decrease Ubc9 protein levels by lowering its phosphorylation status via inhibition of Cdc2 expression.

### Session 8: new concepts of the role of the sinusoidal cells (liver stroma) in primary and metastatic cancer

In the last session of this meeting, the first invited lecture was given by Tania Roskams (University of Leuven, Leuven, Belgium), who introduced the concept of liver cancer cells and the stem cell niche. Hepatic progenitor cells are capable of differentiating towards the biliary and the hepatocytic lineage. An increase in the number of progenitor cells and differentiation towards hepatocytes is a component of all liver diseases. HCC show a range of hepatocellular characteristics, but also progenitor cell features. HCC expressing cytokeratin 19 (CK19), a potential marker for progenitor cells, have a worse prognosis.

Samuele De Minicis (Università Politecnica delle Marche, Ancona, Italy) demonstrated the role of fibrosis as a cofactor in a new mouse model characterized by the evolution of NASH to liver cancer. The authors created a mouse model reproducing the pathological axis of human NAFLD-NASH-cirrhosis-HCC and investigate the role of fibrosis as a cofactor in promoting HCC. WT mice were fed with a CDAA-defined diet and injected with a low dose of CCl_4 _to stimulate fibrogenesis. After treatment, 95% of CDAA+/CCl4- mice revealed tumors and angiogenic/tumor markers were upregulated. CCl_4 _in combination with a CDAA diet induces HCC formation in all animals without changing fibrosis levels.

Another *in vivo *model was used by Le Thi Thanh Thuy (Osaka City University, Osaka, Japan), which demonstrated the promotion of liver and lung tumorigenesis in diethylnitrosamine (DEN)-treated cytoglobin (Cygb)-deficient mice. First, a *Cygb*-KO mouse was generated and treated with *N*,*N*-diethylnitrosamine. All *Cygb*^+/- ^and *Cygb*^-/- ^mice developed DEN-induced liver and lung tumors whereas lung tumors were present only in *Cygb*-deficient mice. Loss of Cygb was associated with increased cancer cell proliferation, overexpression of proinflammatory markers, and hepatic collagen accumulation. Cygb-deficient mice also exhibited increased nitrotyrosine formation and dysregulated expression of cancer-related genes.

Taro Takami (Yamauchi University Hospital, Yamaguchi, Japan) reported that frequent autologous bone marrow cell infusions promote liver regeneration and suppress tumor initiation in hepatocarcinogenic mice with liver cirrhosis. In this study a mouse model of hepatocarcinogenesis with liver cirrhosis (DEN/green fluorescent protein (GFP)-CCl_4 _model) was developed and the kinetics of hepatocarcinogenesis after bone marrow infusion (BMi) was investigated. Foci and tumor in BMi liver showed lower incidence and were smaller in number, while size was almost equal. BMi-treated livers showed significantly reduced liver fibrosis, a lower 8-OHdG levels and a higher SOD activity. Therefore, the authors propose that frequent autologous BMi might contribute to promote liver regeneration and suppress tumor initiation.

The second lecture was given by Matías Avila (University of Navarra, Pamplona, Spain) and discussed the new molecular links between inflammation and liver cancer. Inflammatory mediators and cytokines crosstalk with growth factors and stimulate the survival and proliferation of preneoplastic and transformed cells. For example, epidermal growth factor receptor is activated in acute and chronic liver injury and engages in extensive crosstalk with growth factors, cytokines and inflammatory mediators. Moreover, its ligand amphiregulin (AR) is upregulated in cirrhosis and HCC and conveys important survival and proliferative signals to the transformed cells. Inhibition of the AR/epidermal growth factor receptor (EGFR) signaling system may enhance the efficacy of targeted therapies for HCC.

Bastian Höchst (University of Bonn, Bonn, Germany) reported a new tumor immune suppression mediated by LSEC. The observation that tumors progress in patients with colorectal carcinoma despite the presence of tumor-specific immune responses suggests that tumor development leads to a number of immune suppressive mechanisms. By using different vaccination protocols, tumor models and multicolor flow cytometry, the author showed that LSEC are the dominant cells that take up carcinoembryonic antigen (CEA), crosspresenting it to CD8+ T cells. These tumor-specific T cells show a tolerance-associated phenotype and are less effective in eliminating the CEA-expressing MC-38 tumor cell-line.

## Conclusions

The symposium again demonstrated that research on cells of the hepatic sinusoid is flourishing. The two state-of-the-art lectures, the invited lectures and the excellent work presented as oral presentations or poster presentations reflects the continuing strength of hepatology research in general. Overall, the most relevant aspect emerging from this and the previous meeting of the ISHSR (Pasadena 2010) is the increasing enthusiasm and high level of professionalism of the young participants. Therefore, it is likely that research on the cells of hepatic sinusoids will achieve greater robustness and international recognition. The 17th ISHSR meeting will be held in Osaka, Japan on 23-27 September 2013. Please check the ISHSR website (http://www.ishsr.org) for further information. See you in Osaka in 2013!

## Competing interests

The authors declare that they have no competing interests.

## Authors' contributions

KR and MP contributed equally to this work.

